# Investigating psychosocial stress arising from marker-based optical motion capture and subsequent effects on gait

**DOI:** 10.1038/s41598-025-31814-4

**Published:** 2025-12-11

**Authors:** Sophie Fleischmann, Miriam Kurz, Robert Richer, Luca Abel, Markus Gambietz, Björn M. Eskofier, Nicolas Rohleder, Anne D. Koelewijn

**Affiliations:** 1 Machine Learning and Data Analytics Lab, Department Artificial Intelligence in Biomedical Engineering (AIBE), Friedrich-Alexander-Universität Erlangen-Nürnberg (FAU), 91052 Erlangen, Germany; 2 Chair of Health Psychology, Friedrich-Alexander-Universität Erlangen-Nürnberg (FAU), 91052 Erlangen, Germany; 3 Chair of Autonomous Systems and Mechatronics, Friedrich-Alexander-Universität Erlangen-Nürnberg (FAU), 91054 Erlangen, Germany; 4 Institute of AI for Health, Helmholtz Zentrum München, 85764 Neuherberg, Germany

**Keywords:** Motion capture, Stress, Gait, Marker, Cortisol, Pose estimation, Psychology, Biomedical engineering, Data acquisition, Data processing

## Abstract

Optical motion capture (OMC) requires participants to wear minimal clothing for precise marker placement and involves physical contact with the examiner. We investigate whether the standard OMC procedure induces psychosocial stress in participants and whether it leads to alterations in their gait patterns. Thirty-nine participants took part in a between-groups gait experiment. The OMC group wore short, tight clothing and 39 markers, while the control group wore everyday clothing without markers. Gait was recorded via sagittal-plane video, and gait features (stride times, variability, ranges of motion) were computed from 2-dimensional pose estimation trajectories. Physiological and psychological state changes were assessed using salivary cortisol and self-report questionnaires at multiple time points. Marker placement led to significantly increased negative affect and decreased positive affect among OMC participants, as well as a noticeable but non-significant cortisol response that varied in intensity across individuals. The negative psychological state did not result in significant gait differences on group level, except for late stance knee flexion during slow walking, which may be attributed to reduced pose estimation accuracy due to differences in clothing between groups. Our study suggests that OMC can be used without unwanted gait alterations due to stress at group level. However, contactless measurements that allow participants to wear clothing could alleviate their perceived discomfort.

## Introduction

Precise measurement of human movement is essential in biomechanics. It enables the quantification of a person’s movement and is the basis for subsequent computations of joint angles, velocities, or forces acting during movement^1^. Besides basic research, objective movement analysis has applications in various areas such as clinical evaluations^2^, prosthesis design^3^, or rehabilitation^4^. Regardless of the application, the reliability of movement analysis depends on the accuracy and quality of the collected data.

The current benchmark acquisition method for movement data is optical motion capture (OMC) because of its high temporal and spatial accuracy^5,6^. In OMC, infrared cameras track the motion of reflective markers that are attached to the participant at anatomical landmarks, providing an approximation of the movement of the underlying skeleton^1^. Markersets can comprise more than 43 markers. To minimize the difference between skeleton and marker movement, the markers need to be placed directly onto the skin at bony landmarks^1^. This requires participants to wear little and tight clothing^7^, and involves physical touch by the examiner. Participants remain in minimal clothing throughout the entire experiment, while being continuously observed. While this procedure is necessary to ensure accurate motion data, it also bears the potential of causing considerable discomfort. It has been shown that experimental procedures can cause perceived stress^8^, but it is not known if the standard OMC procedure leads to unintentional psychosocial stress or other related psychological effects among participants.

Psychosocial stress typically arises from social situations that are perceived as uncontrollable or threatening, such as external judgment or social evaluation^9,10^. The body reacts to acute psychosocial stress by activating its internal stress systems, including the hypothalamic-pituitary-adrenal (HPA) axis, which in turn triggers the release of the hormone cortisol^11^. Cortisol levels do not rise immediately but follow a delayed response, reaching their peak approximately 15 to 20 minutes after stress onset^12^. Cortisol can be extracted from saliva, making salivary cortisol a widely used biomarker in stress research^13^. In addition to this physiological reaction, psychosocial stress is accompanied by psychological responses, such as increased feelings of distress, anxiety, or negative emotions, which are typically assessed through self-report questionnaires^14^. Studies have shown that both psychological and physiological stress responses are induced by social-evaluative body image threats in men and women^15,16^. Stress from threats to one’s body image is of particular concern in the context of OMC, where participants are obliged to perform tasks in minimal clothing. We suspect that the usual OMC procedure potentially induces psychosocial stress as well.

Previous research has shown that psychological and physiological states, including stress, are reflected in a person’s posture and movement^17–21^. For example, emotions such as sadness, excitement, or fear, have led to changes in spatiotemporal gait characteristics^22^. With respect to stress, Richer at al.^23^ found that participants moved less under acute psychosocial stress compared to a stress-free control condition, with participants standing in both conditions. Also during walking, Wang et al.^24^ successfully predicted stress severity. These studies indicate that the state of acute psychosocial stress might lead people to involuntarily altering their gait pattern. During OMC, participants should walk naturally to ensure valid and generalizable results. If participants adjust their gait due to potential stress induced by the procedure, this could introduce unwanted effects on subsequent analyses and the conclusions drawn from the experiment.

The goal of our study was to assess whether participants experience psychosocial stress during OMC experiments, which involve marker application and walking with minimal clothing while being under supervision. Additionally, we examine whether individuals undergoing an OMC experiment exhibit altered gait patterns compared to those walking in everyday clothing. We achieve this with a between-groups gait study, during which we measure stress biomarkers and record gait. This paper enables an understanding of the psychosocial impact of OMC experiments and potential effects on the participants’ gait. Our work is particularly important given the widespread use of marker-based motion capture in research and clinical settings.

## Methods

We conducted a gait study with two groups. The OMC group participated in an OMC gait experiment with minimal clothing and markers attached to their bodies, while the control group performed walking in their everyday clothing and without markers. Psychosocial stress was assessed using salivary cortisol and self-reports, while gait patterns were analyzed by extracting features from video data. The following sections provide a detailed description of the experimental procedures.

### Participants

Forty-seven participants without prior experience in OMC experiments were recruited using university mailing lists, flyers, social media and word of mouth. All participants underwent a screening process to ensure their eligibility for the study. Besides OMC experience, exclusion criteria were any physical or medical conditions that might influence HPA axis reactivity (see Supplementary file for detailed list). These criteria were chosen to comply with previous stress studies^23,25^ and guidelines for measuring HPA axis reactivity^26,27^. The study was approved by the ethical committee of the Friedrich-Alexander-Universität Erlangen-Nürnberg (Re.-No. 22-437-B) and conducted according to the Declaration of Helsinki. To minimize the impact of circadian cortisol variaions, all experiments were conducted in the afternoon between 12:30 pm and 6 pm^28^. In addition, participants were instructed not to drink alcohol the day before and on the day of the study, wake up at least three hours before their scheduled session, and avoid strenuous activities and eating for at least one hour prior to the experiment. All participants gave written informed consent before participating.

### Experimental design

**Fig. 1:**
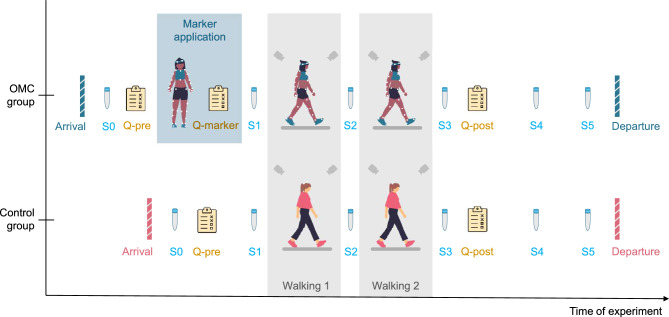
Experimental protocol. Participants underwent a motion capture gait experiment, the optical motion capture (OMC) group with markers, and the control group in their everyday clothing. Six saliva samples were taken during and after the experiment (*S0*−*S5*) and two sets of questionnaires (*Q-pre*, *Q-post*) were filled out at the start and end. The OMC group filled out an additional questionnaire after the marker placement (*Q-marker*).

Each measurement was conducted by two persons, a woman and a man. The participants were randomly assigned to the OMC or control group, stratified by gender. Since only the OMC group walked with markers, the experimental protocol differed slightly for the two groups (Fig.1).

For both groups, a baseline saliva sample (*S0*) was taken upon arrival and after providing consent, and the participants completed the first set of questionnaires (*Q-pre*).

After this, the OMC group was asked to get changed, since they were required to wear only short, tight shorts and a bra if desired, as usually done in OMC studies. This was followed by the marker application, which lasted around 20-30 minutes and was done by the experimenter who identified with the same gender as the participant. We used the full-body Plug-in Gait markerset consisting of 39 markers^29^. After another set of the same two questionnaires (*Q-marker*) that were included to assess the psychological effect of the marker application procedure, the OMC group proceeded to the walking part. In contrast, the control group wore their everyday clothes throughout the entire experiment and walked without markers, which is why they proceeded directly to the walking part after providing baseline sample *S0* and completing the first questionnaire set *Q-pre*.

In the walking part, all participants walked twice on an instrumented dual-belt treadmill (GRAIL, Motek Medical, the Netherlands) for four minutes each: once at a speed of 0.9 m/s and once at 1.2 m/s. During walking, we recorded video data in the sagittal plane at 50 Hz with fixed cameras perpendicular to the treadmill, and ground reaction force (GRF) data at 1000 Hz. The laboratory was equipped with 12 infrared cameras, but since the control group did not wear markers, we did not record marker data for either group. However, to create an atmosphere like in normal OMC experiments, the participants did not know the motion capture cameras were not recording. For both groups, saliva samples were taken before (*S1*), between (*S2*), and after (*S3*) the walking bouts.

After completing the walking part, all participants filled another set of questionnaires (*Q-post*). Subsequently, in the OMC group, the markers were removed, and the participants changed back into their normal clothes. We took two more saliva samples from both groups, the first one (*S4*) 15 minutes after the last sample *S3*, and the second one (*S5*) another 10 minutes later. After the experiment, all saliva samples were stored at -18° for later analysis in the laboratory.

### Data processing and feature extraction

#### Psychosocial stress

The physiological response to the experiment was quantified from the cortisol concentrations in the saliva samples. Therefore, we transferred all samples to the laboratory of the Chair of Health Psychology at FAU Erlangen-Nürnberg, where they were centrifuged at 2000 g for 10 min before the salivary cortisol levels (nmol/L) were determined in duplicate using a chemiluminescence immunoassay (CLIA, IBL, Hamburg, Germany)^23,30^.

The psychological response to the experiment was assessed using self-report questionnaires. We used the Positive and Negative Affect Schedule (PANAS)^31^ and the Short Stress State Questionnaire (SSSQ)^32^ in their validated German translations^30,33^. The PANAS is a validated mood questionnaire to quantify positive and negative affect, each assessed through 10 items rated on a 5-point Likert scale. Individual items are summed to two subscales (*Positive* and *Negative Affect*) with higher scores indicating stronger positive and negative affect, respectively. The SSSQ is a 24-item stress-sensitive questionnaire designed to explicitly assess stress-related experiences. Each item is rated on a 5-point Likert scale, evaluating six different dimensions (*Distress*, *Worry*, *Confidence*, *Negative Affect*, *Motivation*, *Self-evaluation*). Higher scores on each subscale reflect greater levels of the respective factor, and a total score represents the overall perceived stress.

Cortisol levels were adjusted for the baseline by subtracting the cortisol level of baseline sample *S0* from all other samples *S1*-*S5*. In addition, we computed the maximum cortisol increase with respect to *S0* for every participant. From the PANAS, we extracted the *Positive* and *Negative Affect* scores, and from the SSSQ the subscale scores for *Distress* and *Self-evaluation*, and the total score.

#### Gait

We applied 2-dimensional pose estimation to the sagittal plane videos of the walking trials (left lateral view). We used the RTMO (real-time multi-person one-stage) model^34^ within the open-source pose-estimation toolbox MMpose^35^ to estimate the location of 17 anatomical keypoints for every frame: nose, neck, left and right ears, eyes, shoulders, elbows, wrists, hips, knees, and ankles. All trajectories were filtered using a second-order Butterworth filter and a 10 Hz cutoff frequency. The first 30 seconds of each trial were excluded from the analysis so that participants could familiarize with the treadmill.

Left heel strikes were defined as the frame following the maximum distance between the left ankle and hip, corresponding to a temporal offset of 0.02 s (one frame)^36^. We found that this time point aligned more closely with ground truth heel strikes, as determined from available vertical GRF data, compared to the peak distance itself. The trajectories were subsequently segmented into strides, and each stride was resampled to 101 time points. We did not use the force plate data directly to segment the strides because several participants frequently overstepped onto the belt and force plate of the other foot, and for two participants, GRF data were unavailable due to technical issues.

**Fig. 2:**
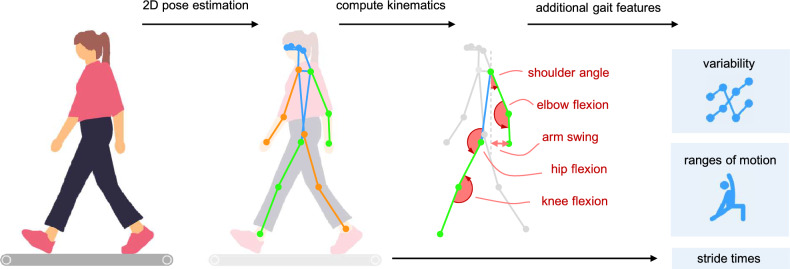
Gait analysis pipeline. We extracted keypoint trajectories from the sagittal gait videos using pose estimation. From the left side keypoints we computed stride times and joint angles, which were then used to extract additional discrete gait features.

Sagittal plane left hip and knee kinematics were computed using Winter’s method^37^. Additionally, we computed the left shoulder and elbow angle, as well as arm swing, as in Kuhtz-Buschbeck et al.^38^ (Fig. 2). For the arms, strides involving non-functional arm movements (e.g., scratching the head or fixing hair) were excluded, identified by a minimal elbow joint angle below 80°.

Stride times were computed as the time between two subsequent heel strikes. Gait variability per trial was assessed by computing the coefficient of variation ($\text {cv}$)^37^ for the hip, knee, elbow, shoulder, and arm swing trajectories:

1 $$\begin{aligned} \text {cv} = \frac{ \sqrt{\frac{1}{N} \sum _{i=1}^{N} \varvec{\sigma }_i^2} }{ \frac{1}{N} \sum _{i=1}^{N} |\textbf{X}_i| } \end{aligned}$$

where $\varvec{\sigma }_i$ and $\textbf{X}_i$ denote the standard deviation and mean over all strides at point $i \in \{1, \dots , N\}$ with $N = 101$ being the number of points per stride. We further computed shoulder, elbow, and arm swing ranges of motion (ROMs). Stride times, coefficients of variation, and ROMs were averaged over both walking bouts for each participant.

### Statistical analysis

We hypothesized that the OMC group would show higher cortisol responses over time compared to the control group. To test this, we conducted a mixed ANOVA with baseline-adjusted cortisol as the dependent variable, sample number as the within-subject factor, and group (OMC vs. control) as the between-subject factor. Where significant effects were observed, pairwise post-hoc comparisons were performed. Additionally, we used pairwise comparisons to assess between-group differences in the maximum cortisol increase.

We further hypothesized that the OMC group would show greater changes in questionnaire scores compared to the control group, and that marker placement would induce an additional increase in perceived stress within the OMC group. For the PANAS and SSSQ, we thus conducted mixed ANOVAs with the respective score as the dependent variable, time (*Q-pre* vs. *Q-post*) as the within-subject factor, and group as the between-subject factor. *Q-marker* was not included in the group comparison as only the OMC group underwent marker application and *Q-marker* was thus not completed by the control group. To assess the influence of the marker placement procedure, we performed a repeated-measures ANOVA within the OMC group comparing scores across *Q-pre*, *Q-post*, and *Q-marker*. In all cases, significant effects were followed up with post-hoc tests.

For gait analysis, we applied statistical parametric mapping (SPM) to evaluate group differences across the stride for the hip and knee kinematics in slow and fast walking, respectively. Pairwise comparisons were used to analyze group differences in discrete gait features (stride times, coefficients of variation, and ranges of motion). SPM was applied only to the lower limbs, as leg kinematics are more constrained by the walking pattern and thus required a time-resolved analysis, whereas we expected upper limb differences to be reflected in discrete metrics such as ROM.

For all analyses, we assessed normality using the Shapiro–Wilk test. Depending on the data distribution, we used either parametric tests (t-tests) or non-parametric alternatives (Wilcoxon tests) for pairwise comparisons. Statistical significance for all tests was set at $\alpha$ = 0.05. The p-values of all post-hoc tests and pairwise comparisons were Bonferroni-corrected within each feature group. For significant results, we report the effect sizes as Hedge’s *g* for pairwise comparisons and partial eta squared ($\eta ^2_p$) for ANOVA results, along with the corresponding F-statistic and corrected p-values. Non-significant statistical results are provided in the Supplementary file.

## Results

Eight participants were excluded because the experimental protocol was not followed (n=5), because of insufficient saliva provided in the sampling tubes (n=2), due to a baseline cortisol level (*S0*) that was more than three standard deviations above the mean baseline cortisol concentration of the study population^23^ (n=1). Therefore, 39 participants ( aged 24.5 ± 4.7 years) were eventually included in the analysis, from which 19 (7 female, 12 male) belonged to the OMC group and 20 (9 female, 11 male) to the control group.

### Cortisol

**Fig. 3:**
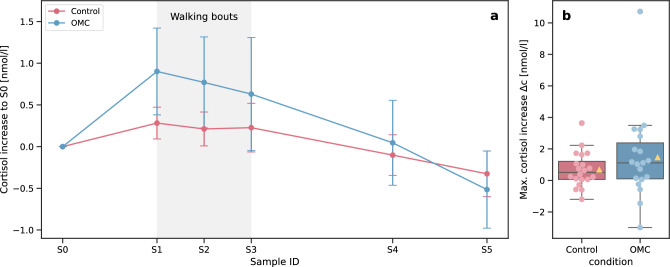
Cortisol analysis results. Left: cortisol change relative to baseline sample *S0* throughout the experiment for OMC and controls. The values are depicted as mean ± standard error per sample ID. Right: maximum cortisol increase for OMC and control groups. The yellow triangle indicates the mean value of the respective condition.

There was no significant difference in the baseline-corrected cortisol response between the groups (F(1, 37) = 0.32, *p* = 0.58, $\eta ^2_p$ = 0.009). The effect of time was significant (F(5, 185) = 6.05, *p* $=$ 0.002, $\eta ^2_p$ = 0.141), but differences were observed only between later samples and not in comparison to the baseline *S0*. We found no interaction effect (F(5, 185) = 1.05, *p* = 0.39, $\eta ^2_p$ = 0.028). Nevertheless, the OMC group did exhibit a more pronounced cortisol response (+0.90 nmol/L from *S0* to *S1*) compared to the control group (+0.28 nmol/L) (Fig. 3). Additionally, OMC participants showed greater response variability (standard deviation of 2.27 nmol/L compared to 0.85 nmol/L for controls). The maximum cortisol increase was also higher in the OMC group with a mean of 1.46 nmol/L (61.5% relative to *S0*), compared to 0.68 nmol/L (37%) in the control group. However, neither the increase from *S0* to *S1* nor the maximum increase was significantly different between the groups. In stress research, it has been proposed to categorize participants as cortisol responders when their cortisol increase exceeds 1.5 nmol/L^39^, which was the case for 7 participants from the OMC group and 4 participants from the control group.

### Questionnaires

**Fig. 4:**
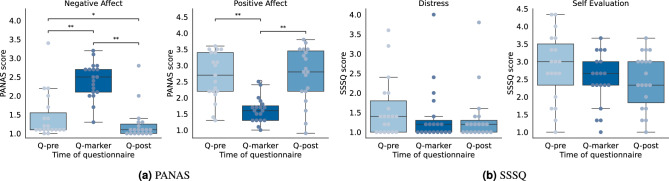
Questionnaire scores from the OMC group at the three assessed time points: experiment start (*Q-pre*), experiment end (*Q-post*), and post marker placement (*Q-marker*). Significant differences are indicated by asterisks (***$p<$0.001, **$p<$0.01, *$p$ $<$0.05). For all scales, the lowest possible score is 1 and the highest possible score is 5.

The repeated measures ANOVA for the OMC group showed a significant effect of time for negative (F(2, 36) = 42.3, *p* < 0.001, $\eta ^2_p$ = 0.70) and positive (F(2, 36) = 31.7, *p* < 0.001, $\eta ^2_p$ = 0.64) affect. Post-hoc analysis showed that the marker placement procedure led to a significant increase in negative affect (*p* $=$ 0.005, *g* = 1.71) and decrease in positive affect (*p* $=$ 0.001, *g* = −1.73) in the OMC group (Fig. 4a). However, at *Q-post*, both scores returned toward their initial values, with negative affect even being significantly lower than at the start (*p* = 0.049, *g* = −0.37). For the SSSQ, there was a main time effect for *Self-evalation* (F(2, 36) = 5.64, *p* $=$ 0.007, $\eta ^2_p$ = 0.24) and the total score (F(2, 36) = 4.72, *p* $=$ 0.032, $\eta ^2_p$ = 0.21). However, while all three scores decreased on average, no score was significantly lower after marker placement in the post-hoc analysis after applying the Bonferroni correction (Fig. 4b).

When comparing OMC and control groups across the beginning (*Q-pre*) and end (*Q-post*) of the experiment, we found no significant group or interaction effect for any of the questionnaire scores. However, we found a main effect of time for the PANAS *Negative Affect* (F(1, 37) = 17.65, *p* < 0.001, $\eta ^2_p$ = 0.32) and the SSSQ *Distress* (F(1, 37) = 6.11, *p* $=$ 0.018, $\eta ^2_p$ = 0.14), *Self-evaluation* (F(1, 37) = 20.15, *p* < 0.001, $\eta ^2_p$ = 0.35) and total score (F(1, 37) = 14.91), *p* < 0.001, $\eta ^2_p$ = 0.29). Post-hoc tests showed that negative affect was significantly lower at the end of the experiment (*p* $=$ 0.001, *g* = −0.48), as were the *Self-evaluation* score (*p* $=$ 0.002, *g* = −0.53) and the total score (*p* = 0.005, *g* = -0.39) from the SSSQ.

### Gait

**Fig. 5:**
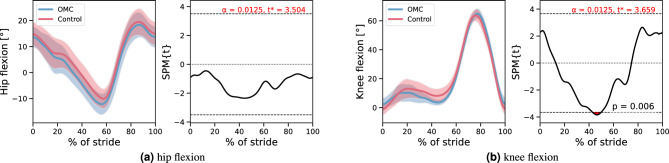
Hip and knee flexion trajectories of both groups for slow walking (left side of each subplot, plots shows mean and standard deviation), as well as the corresponding SPM result (right sides of each subplot). The critical threshold is denoted by the dashed line. The area in which SPM{t} exceeds the threshold is considered significantly different between groups. The corresponding plots for fast walking are in the Supplementary file.

**Fig. 6:**
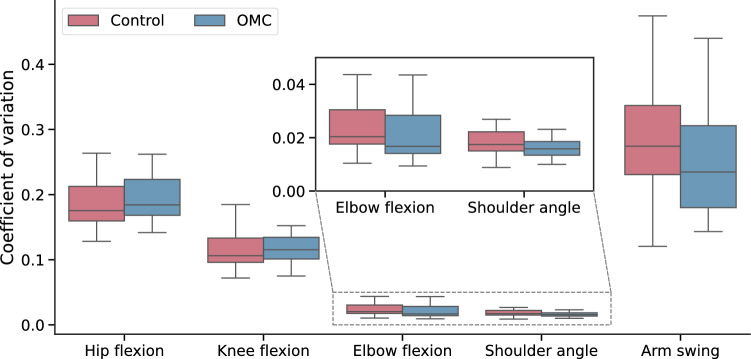
Gait variability features expressed through the coefficient of variation for OMC and control group. Outliers are excluded from the plot for better visibility, but included in the analysis. The same plot with outliers is included in the Supplementary file.

**Fig. 7:**
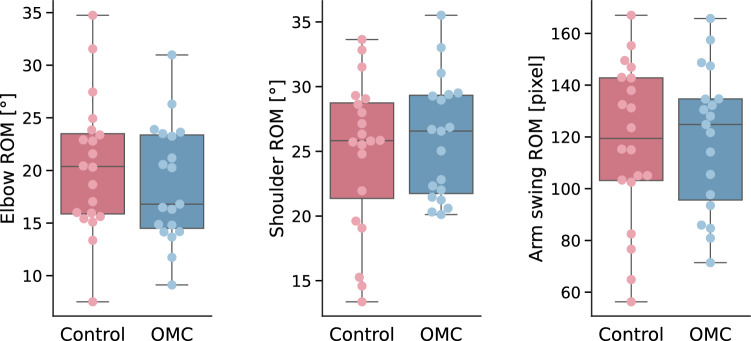
Upper body ranges of motion (ROMs) for OMC and control group.

Stride times were similar between groups (1.19 s ± 0.07 s vs. 1.16 s ± 0.07 s). We found no significant differences in hip kinematics during slow and fast walking, and in knee kinematics during fast walking through SPM. Only for the knee kinematics during slow walking a small cluster between 43% and 50% of the stride exceeded the critical threshold, indicating a significantly lower knee flexion angle during this late stance phase for the OMC group (Fig. 5). We did not find any significant group differences in the coefficients of variation. Nevertheless, the control group tended to show greater arm motion variability, most notably in the arm swing coefficient of variation, with a higher median (0.27 vs. 0.23 for OMC), and higher first and and third quartiles, indicating an overall upward shift in the distribution (Fig. 6). We found no significant ROM differences between OMC and controls (Fig. 7).

## Discussion

In this study we investigated whether participants of a standard motion capture gait experiment experienced psychosocial stress, and whether this affected their gait patterns. OMC is commonly used for human movement analysis, but neither its psychological impact, particularly related to necessary procedures such as undressing and physical contact during marker placement, nor its potential effect on the participants’ gait, have ever been assessed. If participants altered their walking patterns due to stress involuntarily induced by the OMC procedure, this could have influenced the findings of many previous OMC studies. We found that the marker placement procedure induced significant negative affect and significantly reduced positive affect among the OMC group, but we did not find significant differences in other physiological or psychological stress biomarkers compared to a control group who did the same experiment in their everyday clothing. The majority of assessed gait parameters were also not significantly different between the groups.

Although not significant, a trend was visible that the OMC group exhibited a higher cortisol response compared to the controls, especially at sample *S1*. The interval between *S0* and *S1* was shorter for the control group, potentially limiting the detection of the delayed cortisol response at *S1*. However, no increase was observed in later samples either. While the cortisol increase of the OMC group was considerably lower than cortisol responses observed in acute laboratory stress induction protocols, such as the Trier Social Stress Test^40^ (mean increase of >4 nmol/L^40^), it still indicates a physiological response of the HPA axis. Since this axis is highly responsive to social-evaluative threats, we conclude that at least for some participants, motion capture did induce a mild form of psychosocial stress, potentially triggered by a perceived threat to their social-evaluative body image^15,16^. The descriptively higher cortisol response that we observed may be even more pronounced in individuals with greater body dissatisfaction than the participants of our sample, who were mostly young and lean, and free of known health conditions. In our sample, we found no correlation between cortisol increase and body dissatisfaction, as measured by the Body Esteem Scale^41^ with subscales Weight, Appearance, and Attribution. We also observed no significant differences in responses between men and women. The fact that the cortisol levels of both groups dropped below baseline after the walking tasks suggests that the anticipation of the experiment itself may have induced a certain level of tension and activation of the HPA axis among the participants.

The psychological effect of the OMC experiment becomes particularly evident from the PANAS questionnaires. The PANAS, however, assesses affective states rather than stress directly, whereas the SSSQ specifically targets stress-related responses. The high negative and low positive affect scores after marker placement thus indicate that the procedure triggered a shift toward negative emotional states among participants. However, this negative psychological state did not persist after the walking bouts, possibly because participants either habituated to the new situation or became less focused on it as they engaged in another task. The fact that the SSSQ scores did not change significantly is likely because the SSSQ is primarily sensitive to moderate-to-severe stressors^30^, rather than mild psychosocial stress. However, the decrease in the SSSQ scores, even though not significant, also indicates that marker attachment made individuals feel uncomfortable.

The only gait parameter that exhibited a significant difference between OMC and the control group was the knee flexion during the late stance phase in slow walking. However, this finding should not be overinterpreted, especially since the critical threshold was exceeded only slightly. While it cannot be ruled out that OMC participants walked with less knee flexion, it is more likely that this difference stems from our experimental setup. Pose estimation from video camera data is per se less accurate than optical motion capture^42^. A gait analysis study using a camera setup similar to ours reported mean absolute errors of 4.0° and 5.6° for the hip and knee angles, respectively^36^. The fact that in our study one group walked with motion capture clothing and the other group with normal clothing may have introduced additional error. Recent work has reported peak variations between tight and loose clothing of up to 4.48° and 3.82° for hip and knee angles, respectively, using a markerless 3D Theia gait analysis system^43^. The interpretation that part of the observed variability may be attributable to clothing-related effects rather than physiological differences is further supported by our finding of no significant differences in knee flexion between the eleven cortisol responders (those with a cortisol increase greater than 1.5 nmol/L) and the remaining participants. We therefore argue that marker-based motion capture can be used without inducing unwanted gait alterations at the group level.

Nevertheless, we also found trends in the gait features, specifically in the upper body coefficients of variation, where the OMC group showed reduced variability for elbow and shoulder flexion, and particularly for arm swing. We can, however, not determine whether the reduced variability was a result of perceived psychosocial stress, or because the participants walked more carefully to prevent the markers from falling off. Additional analysis could elucidate this by comparing the gait of responders and non-responders, independent of the respective group.

The high variance in cortisol increase in the OMC group shows that the OMC experiment affected participants differently. Not controlling for menstrual cycle phase, which can affect salivary cortisol reactivity^44^, may have additionally suppressed cortisol responses in OMC participants. Due to the between-subject design of our study, we cannot determine how stress affected the gait pattern of an individual. A between-subject design was necessary in our work to ensure that all participants were naïve to motion capture procedures, thereby avoiding habituation or expectation effects. Nevertheless, the effect of stress on an individual’s walking pattern should be investigated using established stress induction methods^40^ in a within-subject setup. This is important because, even though we found no significant gait changes at group level with our between-subject design, it remains possible that an OMC experiment and related psychosocial stress cause unwanted gait alterations on an individual level.

We cannot rule out that the absolute cortisol peak of an individual may have been missed due to the low temporal resolution of our saliva sampling protocol. In addition, the absolute time between S0 and S1 varied depending on the duration of marker application and questionnaire completion. However, if a meaningful cortisol response had occurred before S1, we would still expect to observe corresponding changes in the S1 sample, since cortisol levels typically remain elevated for several minutes after reaching their peak^45^. Furthermore, for all participants in the OMC group who showed an increase greater than 1.5 nmol/L, this increase was observed in more than one sample. It is also possible that a delayed cortisol response might have been attenuated by the counteracting effect of walking and therefore missed. Nevertheless, we do not consider this a limitation, as the experiment was designed to closely resemble an actual motion capture session, during which participants should ideally not experience stress. Moreover, one of our main goals was to assess whether participants alter their gait in response to potential stress, and this interpretation would not change even if a delayed cortisol peak had been masked.

The interaction between participants and examiners may have influenced the participants’ stress response. A previous study using the Trier Social Stress Test reported higher cortisol increases when participants were evaluated by an opposite-sex panel compared to a same-sex panel^46^. Our approach of having a same-sex examiner perform the marker placement and lead the experiment was therefore chosen to minimize potential confounding effects related to the examiner’s sex. Nevertheless, it remains unclear how the results might differ if both examiners were of the same sex or if the opposite-sex examiner had conducted the marker application. In addition, our participant group was relatively homogeneous due to the exclusion criteria, which we selected based on previous literature to minimize potential confounding influences on HPA-axis reactivity. As a result, our findings may not generalize to broader populations, such as older adults or individuals with neurological conditions like stroke, who may also undergo motion capture to track their rehabilitation progress, for example.

In summary, we showed that optical motion capture, specifically the marker placement procedure, led to significant negative psychological states among participants, as well as a notable but non-significant physiological stress response, with differences in intensity between individuals. While we can not directly confirm that OMC induces psychosocial stress, we can conclude that it does lead to perceived discomfort and negative emotional responses. The OMC group tended to have decreased upper body motion variability, the perceived discomfort did not lead to significant gait differences on group level. Nevertheless, contactless motion capture or sensor technologies, that can be used with clothing and would make marker placement obsolete^47^, may help alleviate the psychosocial stress experienced by participants.

## Supplementary Information

Supplementary Information.

## Data Availability

All data needed to reproduce our results is available on Zenodo: https://doi.org/10.5281/zenodo.15721141. The source code for data processing and for all analysis and figures is available on GitHub: https://github.com/empkins/stressgait-analysis.
