# Supplementary material for: Investigating psychosocial stress arising from marker-based optical motion capture and subsequent effects on gait
Source: Sci Rep. 2025 Dec 11;15:43588. doi: 10.1038/s41598-025-31814-4 (PMC12699028; doi:10.1038/s41598-025-31814-4)
Supplement: Supplementary file 1 — Supplementary Information. [file 41598_2025_31814_MOESM1_ESM.pdf]

S1 Participant Exclusion Criteria and Instructions

Exclusion criteria :

- Age <18 or >40 years
- BMI <18 or >30
- Hormonal contraception
- Pregnancy
- Regular drug use or smoking more than 5 cigarettes a day
- Experience with motion capture experiments
- Pathologies: Any malfunction of the nervous system, visual system, cardiovascular system, digestive system; known inflammation; diseases related to liver, gall bladder, pancreas, skeleton, muscles, connective tissues, kidneys, urinary tract, genital organs, blood; any infectious or parasitic disease; cancer; mental disorders
- Intake of specific medication: e.g. for the treatment of high blood pressure, heart disease, cardiac arrhythmias, heart attacks; any anticoagulant or anti-inflammatory medication; insulin, pain medication, anti-depressants, psycho-stimulants, hormones, cholesterol-lowering medication

To minimize the impact of circadian cortisol variaions, all experiments were conducted in the afternoon between 12:30pm and 6pm[1]. In addition, participants were instructed not to drink alcohol the day before and on the day of the study, wake up at least three hours before their scheduled session, and avoid from strenuous activities and eating for at least one hour prior to the experiment.

S2 Additional plots

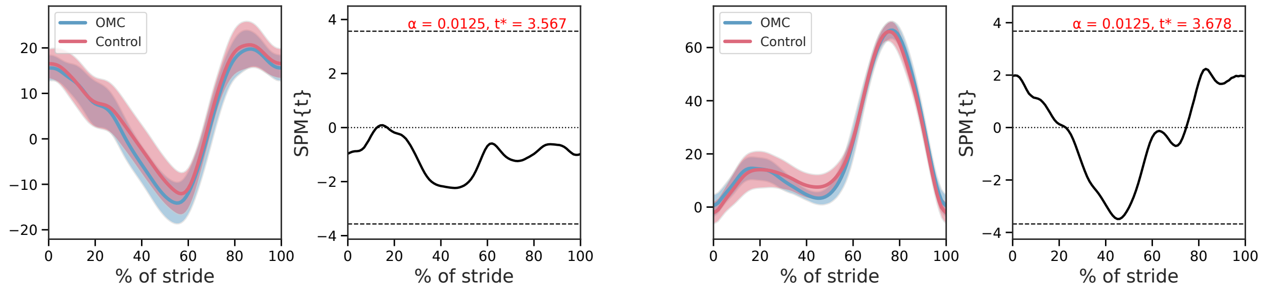

(a) hip flexion **(b)** knee flexion

Figure 1. Hip and knee flexion trajectories of both groups for fast walking (left side of each subplot, plots shows mean and standard deviation), as well as the corresponding SPM result (right sides of each subplot). The critical threshold is denoted by the dashed line. The area in which SPM{t} exceeds the threshold is considered significantly different between groups, which is never the case here.

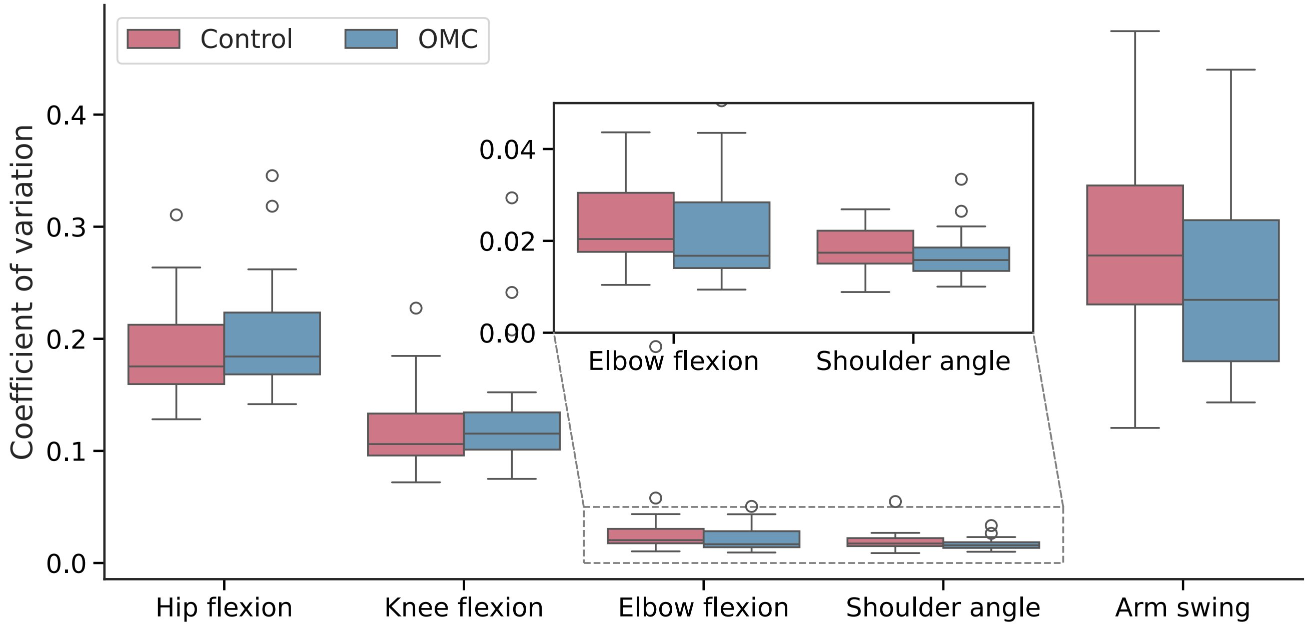

Figure 2. Gait variability expressed though the coefficient of variation. The plot is the same as in the main manuscript, but shows outliers. For the statistical analysis, the outliers were always included.

S3 Non-signigicant statisitical test results

In the following, similar to the main manuscript, we report the effect sizes as Hedge's g for pairwise comparisons and partial eta squared (η_p^2) for ANOVA results, along with the corresponding F-statistic and corrected p-values.

S3.1 Cortisol

- paired t-test maximum cortisol increase: p = 1.0, g = -0.37.
- paired t-test increase S0-S1: p = 1.0, g = -0.36.

S3.2 Questionnaires

Repeated-measures ANOVA (OMC):

- main time effect distress score: F(2, 36) = 2.51, p = 0.12, η_p^2 = 0.12.
- post-hoc pairwise tests self-evaluation:
  - *Q-marker* vs *Q-post*: p = 1.0, g = 0.04
  - *Q-marker* vs *Q-pre*: p = 0.15, g = -0.40
  - *Q-post* vs *Q-pre*: p = 0.33, g = -0.43
- post-hoc pairwise tests total score:
  - *Q-marker* vs *Q-post*: p = 0.43, g = 0.12
  - *Q-marker* vs *Q-pre*: p = 1.0, g = -0.22
  - *Q-post* vs *Q-pre*: p = 0.18, g = -0.34

Mixed-ANOVA:

- SSSQ
  - main group effect Distress: F(1, 37) = 0.34, p = 0.56, η_p^2 = 0.009.
  - main group effect Self-evaluation: F(1, 37) = 0.26, p = 0.61, η_p^2 = 0.007.
  - main group effect total score: F(1, 37) = 0.13, p = 0.73, η_p^2 = 0.003.
  - interaction effect Distress: F(1, 37) = 0.10, p = 0.73, η_p^2 = 0.003.
  - interaction effect Self-evaluation: F(1, 37) = 0.51, p = 0.48, η_p^2 = 0.01.
  - interaction effect total score: F(1, 37) = 0.02, p = 0.88, $\eta_p^2 < 0.001$.
  - post-hoc pairwise test distress *Q-pre* vs *Q-post*: p = 0.19, g = -0.33
- PANAS
  - main group effect Negative Affect: F(1, 37) = 0.19, p = 0.67, η_p^2 = 0.005.
  - main group effect Positive Affect: F(1, 37) = 2.09, p = 0.16, η_p^2 = 0.05.
  - interaction effect Negative Affect: F(1, 37) = 0.009, p = 0.92, $\eta_p^2 < 0.001$.
  - interaction effect Positive Affect: F(1, 37) = 0.71, p = 0.41, $\eta_p^2 = 0.019$.
  - main time effect Positive Affect: F(1, 37) = 0.29, p = 0.60, η_p^2 = 0.008.

S3.3 Gait features

Gait variability paired tests controls vs OMC:

- hip flexion: $p = 1.0$, $g = -0.20$
- knee flexion: $p = 1.0$, $g = -0.30$
- elbow flexion: $p = 1.0$, $g = 0.36$
- shoulder angle: $p = 1.0$, $g = 0.37$
- arm swing: $p = 1.0$, $g = 0.33$

ROMs paired tests controls vs OMC:

- arm swing: $p = 1.0$, $g = -0.04$
- elbow flexion: $p = 1.0$, $g = 0.28$
- shoulder angle: $p = 1.0$, $g = -0.19$

Stride times control vs OMC: $p = 0.17$, $g = -0.43$

References

1. Smyth, J. M. *et al.* Individual differences in the diurnal cycle of cortisol. *Psychoneuroendocrinology* **22**, 89–105, DOI: 10.1016/S0306-4530(96)00039-X (1997).
